# Retraction Note: Uterocervical angle versus cervical length in the prediction of spontaneous preterm birth in women with history of spontaneous preterm birth: a prospective observational study

**DOI:** 10.1186/s12884-025-08158-y

**Published:** 2025-09-05

**Authors:** Ahmed Mohammed Elmaraghy, Salma Mohamed Ahmed Shaaban, Mohammed Salah Elsokkary, Ibrahim Shazly Mohamed Amen Elshazly

**Affiliations:** 1 Department of Obstetrics and Gynecology, Faculty of Medicine, Ain Shams University, Cairo, Egypt; 2 Alexandria specialized hospital, Ministry of health, Alexandria, Egypt

**Retraction Note: BMC Pregnancy and Childbirth 23, 658 (2023)**

**https://doi.org/10.1186/s12884-023-05977-9**

The Editor has retracted this article. After publication, concerns were raised regarding the timeline of the study. The authors were able to explain some of the issues, but did not provide evidence that ethics approval had been received prior to the commencement of the study. Ahmed Mohammed Elmaraghy did not state explicitly whether he agrees to this retraction. Salma Mohamed Ahmed Shaaban, Mohammed Salah Elsokkary and Ibrahim Shazly Mohamed Amen Elshazly did not respond whether they agree to this retraction.

